# A qualitative approach to experiential knowledge identified in focus groups aimed at co-designing a provocation test in the study of electrohypersensitivity

**DOI:** 10.1080/07853890.2022.2114605

**Published:** 2022-09-22

**Authors:** Jimmy Bordarie, Maël Dieudonné, Maryse Ledent, Nicolas Prignot

**Affiliations:** a Qualipsy, EE 1901, Université de Tours, Tours, France; b Pôle de Santé Publique, Hospices Civils de Lyon/Department of Public Health, University Hospital of Lyon; Centre Max Weber, Institut des Sciences de l'Homme, Lyon, France; c Sciensano, Risk and Health Impact Assessment, Bruxelles & Université Libre de Bruxelles, École de Santé Publique, Brussels, Belgique; d Université Libre de Bruxelles, Groupe d'Études Constructivistes, Brussels, Belgique

**Keywords:** Contested illnesses, electromagnetic hypersensitivity, IEI-EMF, qualitative research, participatory research, patient involvement, focus groups

## Abstract

Patients’ experiential knowledge is increasingly recognised as valuable for biomedical research. Its contribution can reveal unexplored aspects of their illnesses and allows research priorities to be refined according to theirs. It can also be argued that patients’ experiential knowledge can contribute to biomedical research, by extending it to the most organic aspects of diseases. A few examples of collaboration between medicine and patient associations are promising, even if there is no single, simple methodology to apply. This article provides feedback on a project involving the experiential knowledge of electrohypersensitive persons with a view to developing an experimental protocol to study their condition. It presents the participatory approach with focus groups that was implemented and reflects on ways to take advantage of experiential knowledge. It also demonstrates the complexity of the electrohypersensitivity syndrome and reflects on the difficult transition between the experiential knowledge and the experimental design of provocation studies.

**KEY MESSAGES**
- Experiential knowledge is a valuable source of information for research and the design of investigation protocols.
- The participatory approach allows co-designing protocols by drawing on experiential knowledge.
- The controversial dimension of EHS reveals the complexity of translating experiential knowledge into an experimental protocol.

## 1. Introduction

Over the last 40** **years, the status of patients has changed, sometimes under the pressure of very active patient associations [1]. Today, patients are increasingly recognised as informed, autonomous, and competent actors. As summarised by Prior [2], “*as the 1990s developed, a concern with belief had been transposed into a concern with knowledge […] and lay people had metamorphosed into multi-skilled and knowledgeable individuals.*” This evolution was made especially apparent by the development of an interest in patients’ experiential knowledge, i.e. which they acquire through immediate and intimate experience of illness rather than through abstract learning [3].

Most attention initially fell on “expert patients,” who have implemented effective techniques to cope with their chronic illness and can teach less seasoned or successful persons. Such experienced patients serve as auxiliaries for health professionals, to whom therapeutic education and the daily management of illness could be delegated, without calling into question the primacy of biomedical knowledge [4]. In that perspective, experiential knowledge becomes a resource to enhance patients’ self-management and self-care. Secondly, the idea emerged that patients or their organisations could make a positive contribution to medical research, supporting and directing it according to their priorities, as in the paradigmatic cases of AIDS [5] and breast cancer [6]. Their experiential knowledge allows them to identify areas of “*undone science*” [7] and underlies a specific mode of engagement that has been developing since the 1990s, referred to as “evidence-based activism” [8]. A more ambitious hypothesis was finally put forward: patients could be in a position to not only influence biomedical knowledge from the outside, by defining research agendas, but also from the inside, by extending it to the most organic aspects of disease [9]. Today, patient associations are doing all three at the same time, as seen for example, in the work of the Huntington's disease patient associations or that of haemophiliacs [10].

### 1.1. Experiential knowledge

Experiential knowledge is a form of specific, non-experimental knowledge based “*on direct contact with realities and phenomena*” (our translation of 11]. In the specific biomedical context, experiential knowledge can be defined as “*knowledge drawn from experience of people's health problems, their knowledge of the trajectory of care and services and the impact of these problems on their personal lives and those of their relatives*” [12]. It constitutes a “*real parallel cultural system*” [13] which cannot therefore be reduced to a mere component of scientific and medical knowledge. Experiential knowledge mobilises multiple resources to deal with diseases [14], which goes beyond scientific conceptions of the disease. Indeed, these resources take the form of multiple knowledge (technical, scientific, traditional, cultural, secular, practical) which Barbier [15] sub-divides into “*theoretical knowledge*” and “*action knowledge*”. Some refer directly to “*embodied knowledge*,” i.e. socially situated knowledge that is part of bodily practices and constitutes a “*determining element in the way in which subjects approach their experience*” [16]. Theoretical knowledge is confronted with experiential knowledge when the former is unable to account for the reality experienced by patients or to answer their questions. Experiential knowledge thus plays a role in the relationship of individuals to diseases in general and thus makes it possible to understand the acceptance or reluctance of certain scientific discourses by patients themselves, sometimes explaining their organisation into evidence-based activist groups [8]. As a result, experiential knowledge appears to be complementary to scientific and biomedical knowledge, justifying the ad hoc inclusion of patients in biomedical research, either for scientific or for medical support [17–19]. It should be mentioned, however, that these examples concern diseases for which a diagnosis can be made.

### 1.2. Electrohypersensitivity

The question arises as to whether such a contribution is actually possible in the context of medically unexplained syndromes, such as Idiopathic Environmental Intolerance attributed to electromagnetic fields (IEI-EMF), often described as electrohypersensitivity (EHS), where considerable controversy surrounds its origin. EHS is an emerging condition characterised by the attribution of medically unexplained symptoms to electromagnetic fields (EMF) of anthropogenic origin [20,21]. Its sufferers predominantly report sleep disorders, asthenia, headaches, memory and concentration difficulties, dizziness, musculoskeletal pain, skin conditions and mood disorders, whose origin is attributed to EMF emitted by various devices, including mobile phone base stations and mobile handsets, Wi-Fi routers, cordless phones, household appliances, compact fluorescent and halogen light bulbs, power lines and power transformers and smart metres (e.g. [22–25]). For people complaining of EHS (EHS people), the attribution of their symptoms to EMF is often a long process, accompanied by medical errancy [20]. The process is characterised by an accumulation of consultations with practitioners of different specialities, as well as personal research to reach this self-diagnosis [26]. Indeed, although in recent years several researchers have suggested the use of biomarkers [27–29] or ultrasonic brain tomosphygmography [30,31] in the EHS diagnosis, these methods are not recognised and validated by medical authorities. As a result, the EHS diagnosis remains bound to its attributions by its sufferers [26,32] and still constitutes a “contested illness,” characterised by a complex relationship between experiential and biomedical knowledge [33].

Indeed, in most of European countries, its sufferers are struggling to obtain its legal and medical recognition as a genuine disease [34,35]. Many patient associations and practitioners are fighting for EHS recognition, both politically and scientifically. This is proving difficult to achieve, as current research tends to conclude that the onset of symptoms is not causally related to EMF exposure [26,36]. The majority of studies that tried to establish its purported electromagnetic aetiology used so-called provocation experiments, which consist in methodically exposing EHS people to EMF and observe their reactions. However, the results of these studies are controversial. Some studies obtain some convincing results [37,38], but many of them do not show any particular ability of EHS people to distinguish between real and sham exposure. This feeds another controversy surrounding provocation studies: the relevance of relying on objective rather than subjective measurements [39]. Furthermore, provocation studies often suffer from methodological shortcomings [36,40]. Thus, whatever their outcome, they allow no definitive conclusion to be reached regarding the aetiology of EHS, which impairs the caring of EHS people.

However, despite this, EHS people often highlight the need to prove experimentally the electromagnetic origins of their condition [41]. In several online testimonials, they even describe the identification of their condition as a form of experimental investigation. Indeed, identifying oneself as an EHS person implies determining which EMFs are involved and how one reacts to them, most often through empirical tests that appear to be lay provocation experiments [20,21,41]. However, these tests have limitations that need to be resolved in order to fulfil the necessary conditions for their scientific recognition. EHS people nevertheless question the relevance of the provocation studies that have been conducted so far, claiming that they are not designed in such a way as to trigger and reliably observe their reactions [42,43]. They cite the available studies focussing on a limited range of sources of exposure and artificially generated, which do not seem realistic compared to the diversity of EMF they are exposed to in their daily lives. Likewise, these studies exclusively dealt with acute and independent reactions, neglecting the effects of chronic and cumulative exposures. In summary, these studies are not deemed to be adapted to their real experiences.

### 1.3. Participatory approach

In this perspective, participatory research is a relevant way to consider patients' expertise and experiential knowledge. The focus-group method appears then as a good strategy with a view to allowing a free speech space to express and share their experiences, but also to apprehending as broadly as possible the experiential knowledge of the participants. This method allows participants to exchange information among themselves, to confront and compare this information and to obtain richer information than in individual interviews. The method has been relevant and widely used since the 1980s in the social sciences [44–47], particularly in areas such as health sciences [48,49–52]. This method is considered appropriate in the study of complex and/or un-researched areas [53]. Focus-group appears as a promising method [54] to “*seek knowledge that is on the boundary between scientific and everyday*” [55, p.114]. This formulation highlights the relevance of such a method to consider an articulation between experiential knowledge and scientific knowledge. Moreover, Krueger and Casey [56] assert that the purpose of this method is “*to promote a comfortable atmosphere of disclosure in which people can share their ideas, experiences, and attitudes about a topic*”. During the focus-group, participants can then focus on their social reality [57–59] and share their views, knowledge, and experiences [60]. This empowers participants [61–64] because of their participation in the project as “experiential experts” relative to their experiential knowledge of the topic. They are thus considered as collaborators of the researchers, and not only as “*guinea pigs*” of the study.

### 1.4. Application to the ExpoComm project

All these elements raise the question of whether it is possible to produce provocation protocols that are both scientifically valid and consistent with the way EHS people describe their condition. Such is the rationale beyond the ExpoComm project in which this study is part. This qualitative research thus sets out to provide feedbacks on the participatory approach adopted and the potential for response and reflection it provides in the development of such an experimental protocol, particularly in relation to the complexity of EHS experiences and the difficulty of translating experiential situations into an experimental test. Here we highlight the elements that we retain in relation to the relevance of experiential knowledge in the development of the protocol. As an example, we will focus our analyses on the process that led to the decision on the type of EMF exposure to which they should be exposed, as well as the intervening issues and precautions discussed.

## 2. Materials and method

### 2.1. Definition of objectives

The ExpoComm project had three objectives:

- Include EHS people in qualitative participatory research using focus groups to collect specific information related to their EMF sensitivity and what they consider necessary to include in an ideal provocation test.
- Co-construct this “ideal provocation test” based on this information and incorporating the necessary scientific constraints in order to propose a provocation test that meets all the criteria to be considered relevant.
- Implement this co-designed protocol and test its acceptability by EHS volunteers.

The purpose of this article is linked to the first and second objectives. It is the exchanges resulting from the qualitative approach of the focus groups that interest us here. In the discussion, we will discuss the success and the limits of the participatory approach we have implemented, which provides information on the possibility of concretely integrating experiential knowledge into scientific co-construction processes for measuring EHS. The description of the protocol co-designed and validated by the participants, as well as the analysis of the acceptability of the protocol, are the subject of a separate article and communication respectively [65,66].

### 2.2. Procedure step by step

In order to meet with the objectives of the project, a method of focus group was therefore favoured so that the participants could actively share and synthesise their experience, while minimising the risk of researchers inadvertently imposing their own understanding of their experience. For the same reason, the preparation and moderation of the focus groups was delegated to professional facilitators not belonging to the research team, the Wallonia e-health Living Lab (WeLL) (http://well-livinglab.be/), while the researchers were present only as observers. The chosen approach included five steps:

#### 2.2.1. Constitution of two focus-groups

In accordance with the literature [56], two focus-groups have been created. Each one was composed of 7 EHS people with various profiles.

#### 2.2.2. Organisation of the first meeting for the two focus-groups – free speech for EHS people

The main objective of the first focus-group meeting was to offer a space for free expression and to ensure that each participant can share their opinions. After a round of introductions of each participant and an explanation of the focus group's approach and objectives, the discussions were guided by a single initial instruction, given by the facilitators: “What would be an ideal test for you that would make it possible to test and measure EHS: what, how, when should you be exposed? What type of waves, etc.?”

#### 2.2.3. Organisation of a second meeting one month later – introduction of scientific constraints

Given the numerous exchanges during the first meeting, a second one was organised to introduce the scientific constraints. The procedure was somewhat iterative, since the first meeting was not sufficient to provide the needed material to design a protocol. The main objective of the second focus group meeting was to articulate scientific constraints of provocation tests [36] and experiential knowledge mentioned during the first meeting. This second meeting was semi-structured, using a guide directly built from the elements highlighted during the first focus-group and including the scientific constraints.

#### 2.2.4. Synthesis of the results

Following the analysis of the discussions, the research team wrote a detailed protocol including elements highlighted by the participants, with due consideration for the scientific, technical, and financial constraints.

#### 2.2.5. Organisation of a third meeting – workshop

The main objective of this meeting was double: firstly, to give an overview of the experimental protocol, and to receive opinions, recommendations, validation, and rejection of the participants; secondly to present the “exposure room” that could meet the criteria of all, participants, engineers and researchers.

### 2.3. Method and analysis

Focus-groups were recorded, transcribed and coded. The main themes addressed by participants were thus identified, as well as the shared views and the types of knowledge underlying them. Given the amount of information generated by the exchanges, we chose here to focus on certain aspects of the test. Thus, we will not present all the themes identified, but only the more relevant for the co-designed process.

### 2.4. Participants and recruitment

Recruitment was carried out through several channels: contacts obtained from previous studies or from home exposure measurements, an advertisement on the website of a building biologist, word of mouth… Associations of EHS people contacted to be involved in the process had rejected our proposition, as discussed in a separate publication [67]. They were reluctant to participate in the project due to various concerns, such as fears about the independence of the study and conflicts of interest of the researchers, the specialisations of those involved in the project (social science oriented) and their lack of time to devote to it.

Seven men and seven women were included in the two focus groups (Table 1). The age of the participants was between 38 and 72** **years old (median at 54** **years old). Among the characteristics of the participants, we note a different level of certainty of suffering from EHS among the participants and the attribution of symptoms to EMF for a more or less long time: two people questioned their sensitivity while eight others stated that they had been suffering from symptoms attributed to EMF for at least 10** **years.

**Table 1.** Characteristics of participants (*W_x_*: Woman; *M_x_* : Man).

	Age	Awareness of EHS (*n* years)	Incriminated sources
W1	58	2	RF/50** **Hz
W2	41	Questioning	RF
W3	54	3	RF
W4	63	10	Wi-Fi
W5	64	>10	RF/50** **Hz
W6	72	22	RF
W7	64	Questioning	Mobile phone/Wi-Fi
M1	45	4	RF/50** **Hz
M2	47	>20	RF/50** **Hz
M3	/	>20	RF
M4	55	14	RF/50** **Hz
M5	44	10	RF
M6	38	10	RF/50** **Hz
M7	48	1	Radar/Mobile phone

## 3. Results

Of note, participants reported that they appreciated the opportunity to share their experiences and collaborate on the development of a scientific study on EHS. In general, participants agreed on the great complexity of EHS, stating that *“there are as many forms of EHS as EHS persons*,” the difficulties they encounter in identifying it, and the unpredictability of their reactions. Because of the differences in the way they experience their sensitivity, it is very difficult for participants to agree on unanimous and unambiguous responses regarding many aspects of the protocol. Indeed, the highly individualised expression of EHS-related disorders necessarily leads to consider it carefully to better envisage the integration of experiential knowledge into co-designed processes. For this reason, we have chosen to present the results here by considering the three meetings-stages that we followed within the framework of this project: firstly, a free speech focus group; secondly, a semi-structured focus group using a guide directly constructed from the points highlighted during the first meeting and including the scientific constraints; thirdly, a restitution workshop aiming to present the experimental protocol and to receive the opinions, recommendations, validations and rejections from the participants.

### 3.1. First meeting for the two focus-groups: free speech for EHS people

The results highlighted, from the outset, a heterogeneity of responses regarding the type of EM sources to be used in the protocol: some participants expressed difficulties in defining a specific source due to quasi-permanent symptoms or an inability to identify the sources of exposure that are problematic due to the ubiquity of EM sources in their environment. Indeed, defining the most relevant signals appears to be a particularly complex issue, both because of the extreme variability of wave sources in the environment and the difficulty in identifying the real source potentially causing the symptoms experienced, but also because of the need for participants to imagine a test that is as close as possible to their reality. In other words, the ideal test for detecting EHS appears to be a highly individualised test, considering the specificities of each person. Some comments illustrated it as the following one: “*And maybe your situation or your situation or your situation won't make me sick. That's what EHS is all about, it's hyper-vicious*” (M6). We understand that participants try to provide an answer by relying on concrete situations. They then seem to prefer a representative exposure of “*what* [they] *are exposed to on a daily basis because […] maybe the pure wave* [authors’ note: single frequency, non-modulated; as it could be in case of a signal artificially generated] *would affect us less”* (W2). That explains why some participants are in favour of measurements in places where they are affected, to characterise the waves of interest. Eventually, this led the participants to question the conditions under which they themselves feel able to discriminate between the presence or absence of waves. The discussions between participants underline two distinct periods: one related to the need of reaching a “neutral state before exposure” and one related to “sensitivity during exposure”.

The first period is related to the conditions before the test for which most of the participants quickly agreed on the need to start the test in a state defined as neutral, “*completely emptied* [of the influence of the waves]” (M5) or a state of normal reactivity to the waves. For several of them, this “neutral” state is the only one in which they expressed they would feel able to effectively discriminate between real and sham exposure. Nevertheless, the return to the neutral state is experienced very differently depending on the participants and the necessary length of this rest period varies greatly from one participant to another. Some argued that a good night’s sleep is enough, others talking about a period of several weeks in a wave-free environment to reach it or explaining that perhaps “*a Faraday cage*” (M5) would be the solution. Others experience this neutral state can disappear very quickly: “*You just have to be exposed to a computer for a few minutes, in my case that's it, eh, then it's over. It goes very quickly*” (W6). Others feel more sensitive with fatigue. In addition, several participants stressed that, undergoing a provocation test would force them to face the effects of these EMF and to their sensitivity that they generally seek to numb because it is a source of suffering for them. That mean that they would give up their own “well-being” solutions, e.g. by taking food supplements or other practices. The need to prepare this state of sensitivity is viewed by some participants as a substantial constraint on the possibility of carrying out a scientifically sound provocation test: “*Yes, the problem is that it will result in a study where everyone is in a different situation, so it is not generalizable, so it is not scientific”* (M5). For some, total neutrality “*will be almost impossible to achieve, everyone is in a different state, with their own feelings, with the things they have come across during the day… but on the other hand, perhaps there would be a way of allowing a moment of resourcing before the test*” (M2). A participant stressed that “we *have to accept a certain degree of prior sensitivity, because otherwise it's not possible*…” (W4). The second period concerns sensitivity during the exposure. Stress or anxiety is seen as a disrupter of sensitivity linked to EHS. Participants agree that the testing situation itself can be a source of anxiety, and therefore of failure of the provocation test. The focus is steered towards those elements that could limit anxiety. Discussions did not lead to the definition of EMF exposure of interest in an ideal test, but rather to some characteristics to be considered in the development of the exposure system and the type of waves.

### 3.2. Second meeting: introduction of scientific constraints

So, prior to this meeting, researchers and engineers developed a pre-list of EMF signals that would be acceptable to as many people as possible based on the discussions during the first focus-group, including radiofrequency signals emitted by a Wi-Fi box, a DECT base station, an antenna emitting real 2** **G 3** **G 4** **G signals, and a current loop generating 50** **Hz fields. Solutions related to the conditions required to feel able to discriminate between the presence or absence of waves were also discussed beforehand. The aim of this focus-group was to verify the choices and to see how these signals should be generated, e.g. one signal at a time or in combination. At the beginning of the focus-group the question was first raised in an open format. Then, the facilitator presented the options that are being considered by the scientific team.

The discussion on wave sources mostly led to the same conclusions in both groups: Wi-Fi was first mentioned, followed by radar, mobile phone and tablets. In Group 1, other sources are also proposed, such as radio/TV signals, as well as an unknown signal. One participant points out that he has the impression that a cocktail of waves would better fit his sensitivity (M4). In Group 2, other signals are also suggested, such as neon, microwave, or light frequencies, also Bluetooth diffusers and the hands-free system in the car. Following a participant's comment “*A bit of what we encounter in everyday life, that's the point, right?*” (W6), the discussion turned directly to the form: “Should we be exposed to an isolated wave, or to a ‘cocktail as in a real environment’?” When the facilitators presented the considered signals, the participants agreed with these signals, with the exception of one participant in Group 1 who says, “*For me I will hardly react I am almost certain*” (M1), the same one who would prefer to be exposed to an unknown wave because “*when you get used to them you become less sensitive*”. As for the form of the exposure, opinions diverge. A consensus is not reached at this stage, some favouring a cocktail of signals, others a sequence of exposure to successive waves or even an isolated wave. They agree on the importance of being able to test beforehand to ensure that the exposure is appropriate to their sensitivity.

At the end of the second meeting, fairly fundamental uncertainties remain in the definition of EMF signals of interest and the form of exposure. To avoid researchers getting involved in certain decisions that they intended to leave to the participants, an individual questionnaire was sent to them concerning the pending points of the whole process. Considering EMF exposure, questions were: (1) What types of electromagnetic fields should participants be exposed to? (2) Should participants be exposed to only one or several types of fields? The four signals considered are globally retained by the participants, five of them noting however that they do not feel sensitive to 50** **Hz fields. Two also talk of a radar signal and three others mention the interest of an unknown wave. Concerning the form, except for one participant who would like to be exposed to only one type of wave, they agree on the interest of a cocktail-type exposure.

### 3.3. Workshop of restitution

After confirming the feasibility of the technical choices and ensuring the criteria of scientific quality, the third workshop took place.

This workshop allowed the participants to verify the suitability of the premises and the environment. Each group, in turn, visited the planned test premises. An engineer was present to answer technical questions and confirm the choices made. Regarding EMF exposure, a cocktail of EMF has been proposed, gathering the signals emitted by a Wi-Fi box, a DECT base station, an antenna emitting real 2** **G 3** **G 4** **G signals (signals recorded from an antenna in the neighbourhood), as well as fields generated by a current loop placed in the ceiling. The protocol was welcomed by most participants as all but one agreed with the defined exposure setting.

This participant (M1) reports that he feels more sensitive to electric current than to fields in general. Rather than being exposed to EMF signals as proposed, he would like to test his sensitivity by e.g. holding a cable, whether powered or not, in a double-blind test. No other comments were made about the exposure system.

## 4. Discussion

Related to exposure characteristics, this required, on the one hand, an inclusive approach, with as broad a “cocktail” of EMF as possible and, on the other hand, a certain degree of personalisation (e.g. in the length of the pre-exposure rest and exposure period). All the contributions leading to the co-developed protocol has been published [66]. However, this qualitative study and the use of focus-group to the process of co-construction should undergo extensive discussion, which we will try to address here through several points. Next to the identification of limits of the process, we will focus here on the following questions:

- Has the focus group method proved to be effective in this co-construction process and what can we expect from this process? (4.1 and 4.2)
- How to articulate experiential knowledge and scientific constraints when they are two sides of the same phenomenon with different objectives, the ones seeking to improve the knowledge of EHS, the others to be recognised in their sensitivity? (4.3 and 4.4)

### 4.1. What are the identified limits of the process?

In the co-designed process, we aimed to work with as wide a panel of people as possible, differentiated on the basis of their age, gender and length of recognition as EHS people. For this last characteristic, the difficulty lies in the very definition of the EHS condition, which is self-attributed. One of the main findings of the focus groups was that it was very difficult for the participants to express a common reality and understanding of what constitutes EHS. Indeed, it appears that everyone experiences EHS as something very subjective, whether it is the symptoms, the latency period, the possibility to reach a neutral state or the impact on daily life. This is a key point, which may be a limitation for the future. The great heterogeneity of the realities expressed by the participants is the very manifestation of the difficulty of envisaging scientific approaches that would not consider these elements specific to each EHS person. In our opinion, this confirms the approach of the ExpoComm project, which aims to design an exposure protocol in accordance with the expectations of the people concerned and the specificities of their own feelings about EHS. In this self-attributed, but controversial condition, no objective means are yet validated to ensure the reality of this attribution [26]. At best, as suggested by Szemerszky et al. [68], the impact of sensitivity in daily and professional life could be assessed as an indicator of the severity of the condition. The exploration of relevant biological markers also constitutes an attractive avenue of research to insure the recognition of the EHS condition.

The question on the limits of the process relates to the adequacy of the outputs resulting from the first focus-group and the expectations of the researchers, in particular in view of the complexity of imagining a provocation test which makes it possible to consider all the specificities of EHS people. Overall, the participation of EHS people was invaluable in obtaining the necessary information. They were ready to give their opinion and to try to project themselves in a test situation during the second focus-group. However, participants have revealed such a heterogeneity in their opinions and expectations relative to provocation tests that there was a major difficulty in synthesising this into an ideal test. Although EHS people often follow a form of experimental protocol similar to what would be done in provocation tests [20,21,41], they are not unanimous about the test version to be implemented as we saw for the exposure signals or neutral state to be achieved. At this point, researchers need to ask themselves what such a process can yield.

### 4.2. What to expect from the co-designed process and the focus-group method?

The study of EHS requires an interest in the EHS people themselves in order to understand both their subjective experience, which is expressed in very variable ways, and the social reality they share facing scientists and physicians who do not provide them specific answers to the disorders they suffer. The focus group method appeared to be the only tool available to start from the experience of the participants themselves, to understand the complexity of the connection with their symptoms and to build a protocol that involved them as individuals facing concrete problems and that could be tailored to their needs.

Thus, the focus-group method made it possible to avoid relying on isolated testimonies. But this method also shown certain limitations, which have led us to propose a semi-structured focus-group that had not been foreseen in the beginning of the process. Authors have already cautioned against the use of focus groups, questioning the easiness or the efficiency of such a method [69–72]. Moreover, previous studies have already concluded that the focus group may be less appropriate than hoped for in so-called sensitive research, such as sexuality or abuse for example [73–76]. Lee [74] defines “*sensitive research*” as any study that deals with a subject related to a potential issue such as threat, embarrassment, offence and/or social censure. In the present case, EHS appears to be a sensitive subject in the sense that it is not recognised by biomedicine and scientists as a disease, or at least as a disease whose cause lies in electromagnetic waves, as claimed by EHS people. It has become the subject of considerable controversy in the medical and scientific communities. Therefore, there is a threat for EHS people not to be recognised.

The public and patient involvement nowadays appears as a social practice with a high signification and the role of this involvement for patients goes beyond the health issues [77]. Indeed, beyond their scientific objectives, researchers must focus on participants’ motivations for this kind of collaboration and analyse its consequences. Participants’ motivations in our study are the subject of another publication [67] in which we show how the need for recognition of their disease as such is at the very heart of their implication in EHS research projects and exposure protocols.

These focus groups and what they have generated in terms of results in a certain way question the expectations of the scientists who felt helpless in not initially obtaining the “technical feedback” they hoped for to build their protocol. But here it is actually the scientists' expectations that need to be questioned, not the discourses of the participants. Indeed, as experts in their perceptions of EMF, they were invited to share their feelings about the expression of their symptoms and their views on an ideal test. However, this expert knowledge concerns their experience and perceptions of EHS people, not their technical or statistical knowledge, nor their ability to produce effective laboratory tests. If the involvement of patients or the public in scientific processes appears to be means in restoring a certain democratic deficit in traditional research [78,79], it must nevertheless question the real impact of this contribution [80]. This question, which cannot be avoided [81], must be asked in terms of costs and benefits, both for the research itself and for the contributors [82,83,84]. It must also be asked in terms of the contributors' experience and knowledge [85].

### 4.3. From scientists’ expectations to participants’ experiential knowledge

Thus, researchers’ expectation must itself be discussed, for while it may seem entirely justified, it must nevertheless be questioned on the basis of what we have learned in these focus groups.

The choices pre-determined by the research team are a reminder of this: the scientific constraints are set by the team, and only the modalities falling within these constraints will be understood and translated by the team into a laboratory protocol. It should also be pointed out that the request made to participants during the focus groups, which was not simply to talk about their experience, but to imagine an ideal setting in which they could reveal their sensitivity, is a very abstract request. What is asked to the participants is to imagine a situation that most of them have never experienced, and to conceptually confirm its interest. Although EHS people describe situations where they have tested themselves, it is a sensitivity located in a particular environment, but not necessarily in a laboratory where the parameters are under control. We are talking about patients as experts in their own experience of a health condition, but not about asking patients to become scientific experts of the research field [79,86,87]. This raises questions about the role and consideration attributed to the knowledge of contributors which sometimes need a learning process to demonstrate a form of capital [85]. However, this learning process could lead them to lose both the very nature of the reasons why they are associated with in the co-designed process, but also their credibility with other people likely to experience similar problems to their own [79]. In this study, the lack of scientific learning process could be a reason explaining the difficulty to concretise the co-designed process, even though at the end, a provocation test has been provided taking into account the participants’ contributions and being mostly accepted by the participants.

Scientists’ expectations are generally linked to their own preconceived ideas about the phenomena they study [88,89], but also to their willingness to objectivise phenomena in order to envisage generalisable results. These expectations are an important limitation that refers to the repeated description of many parameters that participants do not control, such as the return to a “neutral” state. Facing this need, EHS people seek to be recognised as sick of the waves and to be considered in their subjectivity of suffering individuals. Results shown that the focus groups can provide them with a place for expression and socialisation [82], and a recognition of the health issues they experience [84]. Indeed, the focus group allowed participants to share personal stories. At the same time, we sought to reflect on an exposure protocol considering their daily experiences. From this perspective, the focus groups were a good method to use. This co-designed project with EHS people contributes to propose new aspects for provocation protocols. However, as it has been said, we can question the legitimacy of such a co-designed protocol due to the extreme heterogeneity of the sensitivities and their manifestations, and the expectations of EHS people. Indeed, the gap between the scientific willingness to objectivising phenomena and the need for people to be considered in their individual characteristics can constitute an important problem; and we can wonder how EHS people who did not participate in these focus-groups could receive and accept this protocol. This is particularly true since the participants in this study are individuals who have accepted, of their own accord, to be part of the research process, but the associations contacted did not want to participate to this project. This is an important limitation because if patient involvement improves the overall research process and its legitimacy [90], it is generally with the agreement and involvement of the associations themselves. In this project, only a few individuals belonging to an association could relay information.

### 4.4. Experiential knowledge, experiment and biomedicine recognition

The analysis of the focus groups shows that the participants’ experience is particularly complex, reinforcing the difficulty of translating the contribution of their experiential knowledge into a provocation test, the limits of which we have already questioned. Participants often used examples in a confirmatory manner, using stories of retrospective discovery of EMF exposure – a line of reasoning known to lead to erroneous correlations (e.g. the example of arthritic pain initially associated by patients with the weather, whose symptom diaries were not correlated with records from nearby weather stations [91]). The links made by our participants to examples of places or circumstances where they had experienced symptoms and suspected or demonstrated the presence of EMFs illustrates the intrinsic limitations of their experiential knowledge, which applies only to phenomena that can be directly experienced. This is based on a change in their health status without actually identifying the causes of these changes [2]. Moreover, in the case of EHS, symptoms are attributed to phenomena that are assumed to be imperceptible to humans, requiring the use of vicarious means to detect EMFs in their environment [92], while the results of these measurements are generally quite imprecise.

Their experiential knowledge remains irreducibly embedded in the singular contexts in which it is produced, hence its presentation as a collection of anecdotes, making it difficult to overcome the diversity of EHS manifestations. It should also be noted that the transformation of a phenomenon into experiential knowledge requires a great deal of invention, and that it is always a matter of creating new ways of relating to a phenomenon [93]. But the difficulty or impossibility of translating a phenomenon into an experimental protocol does not mean that it does not exist.

The fact that EHS, as characterised by sufferers’ attributions, remains deeply labile and idiosyncratic, however, may result not only from the limitations of their experiential knowledge, but also from the medical illegitimacy of their condition. The cognitive strategies of EHS sufferers do not appear to be significantly different from those of other patients, for example with chronic obstructive pulmonary disease [18]. But these patients suffer from a legitimate disease, meaning routinely diagnosed by doctors, which has two notable effects on their experience: on the one hand, it ensures that it comes from similar disease processes and is inherently less disparate; on the other hand, it further homogenises it by framing it in shared medical terms. The knowledge derived from this experience thus becomes more coherent by being “medically socialised” [18]. In comparison, people with contested and self-diagnosed conditions such as EHS have no external support to abstract from the singularity of their experiences. Incidentally, this makes it even more difficult for them to associate: they must first establish that they are suffering from the same illness, by linking their experiences and establishing that they are similar [94]. In other words, biomedicine provides a structuring framework for the experience of illness, which remains particularly difficult to escape from when seeking to integrate patients’ experiential knowledge into biomedical research. Our study raises the question of a time-bound approach to co-construction processes based on patients’ experiential knowledge. If in most cases, experiential knowledge is part of a learning process allowing patients to capitalise [84] on the mechanisms and processes at work in their illness, it is because biomedicine already recognises their illness. EHS poses a new challenge: to successfully integrate experiential knowledge through the involvement of EHS people in order to identify, measure and understand EHS, even before biomedicine is able to recognise it as a disease.

## 5. Conclusion

In summary, the participants’ involvement with the method of focus-groups were appreciated and participants were very cooperative and willing to share their personal experiences and their expectations to provide information to the researchers. The diversity of individual experiences required an inclusive approach that entailed the possibility of individualising test conditions. On this basis, it was possible to elaborate a protocol that achieved a broad consensus among the participants. In addition, the focus-groups were more successful in another respect: this qualitative and participatory approach helped build trust with participants, who deeply appreciated being treated as individuals with a certain expertise relative to their health status, despite the controversy surrounding their condition. The reported limits do not invalidate the value of experiential knowledge as a complement and challenge to researchers, allowing to better address the desires and needs of participants. However, considering the integration of experiential knowledge in the process of scientific construction needs to build a specific agenda related to the objectives of biomedical research, which are very specific for EHS given that, unlike other pathologies, official biomedicine does not recognise it as a disease.

## Data Availability

The data that support the findings of this study are available from the corresponding author, [JB], upon reasonable request.
